# Age-related differences in cerebral blood flow underlie the BOLD fMRI signal in childhood

**DOI:** 10.3389/fpsyg.2014.00300

**Published:** 2014-04-16

**Authors:** Pamela Moses, Leanna M. Hernandez, Elizabeth Orient

**Affiliations:** Department of Psychology, San Diego State UniversitySan Diego, CA, USA

**Keywords:** brain development, perfusion, hemodynamic response, arterial spin labeling

## Abstract

Functional magnetic resonance imaging (fMRI) has become a premiere technique for studying the development and neural mediation of a wide range of typical and atypical behaviors in children. While the mechanism of the blood oxygen level-dependent (BOLD) fMRI signal has been a focus of investigation in the mature brain, it has been largely unexamined in the developing brain. One critical component of the BOLD signal that has been noted to change with age is cerebral blood flow (CBF). Reports of CBF in children based on clinical radioactive tracing methods have found elevated CBF in childhood relative to adulthood, which could affect the BOLD response. This study used non-invasive arterial spin labeling magnetic resonance imaging to study resting state and activity-driven CBF in conjunction with the functional BOLD response in healthy children 8 and 12 years of age and in adults. Participants performed a finger-tapping task to generate robust activation measured in the motor cortex. Quantification of resting state CBF demonstrated higher CBF in 8 year olds and in 12 year olds relative to adults. The absolute increase in CBF between baseline rest and peak response during the motor task was also higher in both child groups compared to adults. In contrast, the relative increase of CBF above baseline, expressed as percent of CBF change, was comparable across groups. The percent of BOLD signal change was also stable across age groups. This set of findings suggests that along with elevated CBF in childhood, other component processes of the BOLD response are also in an elevated state such that together they yield a net BOLD effect that resembles adults. These findings coincide with our previous examination of hemodynamics in primary sensory cortex. Although the magnitude of the BOLD response appears consistent between childhood and adulthood, the underlying physiology and cerebrovascular dynamics that give rise to the BOLD effect differ between immature and mature neural systems.

## INTRODUCTION

Over the last decade, there has been a rapid increase in the number of scientific investigations employing blood oxygen level-dependent (BOLD) functional magnetic resonance imaging (fMRI) to study brain development *in vivo*. The non-invasive nature of the BOLD technique, which allows for the comparison of patterns of brain activity across a wide range of subject populations, is one of the key reasons for its growing popularity. Increasingly, BOLD fMRI has been applied to healthy children and adolescents in hopes of better understanding typical brain development, yet some basic questions about the underlying physiological components of the BOLD signal itself, and possible age-related changes in these physiological parameters, have not yet been addressed.

Cerebral blood flow (CBF) plays a key role in the BOLD signal, since it directly influences proportions of oxygenated and deoxygenated blood in the resting and active brain. The characteristic increase in BOLD signal observed during cognitive stimulation occurs, in part, due to an increase in CBF, a physiological parameter within the brain that is significantly elevated in children during the first two decades of life relative to adult levels (Chugani and Phelps, 1986; Ogawa et al., 1989; Chiron et al., 1992; Kuroda et al., 1993; Barthel et al., 1997; Takahashi et al., 1999). Previous studies have shown that elevated baseline levels of CBF can result in reduced BOLD response (Bruhn et al., 1994; Bandettini and Wong, 1997; Cohen et al., 2002; Brown et al., 2003; Stefanovic et al., 2006; Vazquez et al., 2006). Thus, baseline differences in CBF rates between children and adults have the potential to produce an age-related confound in the BOLD signal, which would be of critical significance to the growing field of developmental neuroimaging.

Our current knowledge of CBF developmental trajectories is limited since previous studies have mostly been conducted in clinical pediatric populations using positron emission tomography (PET) and single photon emission computed tomography (SPECT). Application of PET and SPECT techniques require the injection of radioactive isotopes into the vasculature, where they are carried to the brain via the blood stream. Overall, these studies have reported CBF rates in infancy that increase during early childhood (approximately 3–8 years of age, when CBF peaks at rates 50% higher than adult levels), and then gradually decline during late childhood and adolescence (approximately 10–18 years of age), reaching adult levels in the second decade of life (Chugani, 1987; Ogawa et al., 1989; Chiron et al., 1992; Kuroda et al., 1993; Barthel et al., 1997; Takahashi et al., 1999). In addition to elevated whole brain CBF, regional CBF measured in multiple brain areas (across hemispheres and lobes) also exhibits developmental change with each cortical area maturing at slightly different ages, but overall showing a pattern of elevated CBF in childhood and decreasing levels in adolescence and adulthood (Ogawa et al., 1989; Chiron et al., 1992).

However, there are several limitations to radionucleotide techniques. Since both PET and SPECT expose subjects to low doses of radiation and are invasive, their research applications are limited mainly to clinical pediatric and adult populations. Furthermore, these studies often utilize some degree of sedation, yet the effects of sedative medications on CBF have not been fully investigated and are incompletely understood (Biagi et al., 2007). Lastly, pediatric CBF radionucleotide studies have been conducted while the subject is at rest, without stimulation. Thus, while clinical studies of children at rest suggest elevated CBF in childhood relative to adulthood, we are lacking data on age-related CBF trajectories in typically developing subjects. Whether these differences persist during neural activity remains an open area of investigation.

Arterial spin labeling (ASL) magnetic resonance imaging (MRI) offers an alternative to the invasive methods of radionucleotide techniques and provides a safe approach for measuring CBF *in vivo* during both rest and stimulation. Similar to PET and SPECT, ASL applies a tag to blood flowing into brain; however, instead of injecting a radioactive tag to label ascending blood, ASL applies a transitory magnetic tag. After a delay to allow the magnetically tagged blood to flow into the brain, an image is acquired (the “tag” image). Next, a “control” image is taken of the same brain region without the presence of magnetically tagged blood. The MRI signal values from the tag image subtracted from the values in the control image provide a measure of CBF. ASL pulse sequences can also be optimized to allow for simultaneous acquisition of CBF and BOLD data (Wong et al., 1997). The ability to quantify both blood flow and BOLD changes during rest and activation and the non-invasive nature offered by ASL techniques make ASL an ideal method for investigation of CBF and neural activity in healthy children and adults alike.

Arterial spin labeling studies of developmental change in CBF have largely supported previous findings produced with PET techniques. They provided further evidence of increases in CBF after birth that peak during childhood and decrease in adolescence until reaching adult levels (Wang et al., 2003a,b; Biagi et al., 2007). Notably, the age of peak CBF varies by gray matter region, with primary areas peaking before cortical association areas (Taki et al., 2011). In comparing children aged 4–12 years to adults aged 20–78 years, Biagi et al. (2007) found a 40% elevation in CBF in the children compared to adults. However, the children in this study were initially referred for clinical MRI scans (later determined to be neurologically normal), which may not be representative of typical development, and a subset of the children were imaged while under sedation.

A limited number of studies have examined characteristics of the hemodynamic response in children. Richter and Richter (2003), Kang et al. (2003) and Wenger et al. (2004) conducted fMRI studies with sensorimotor tasks to measure and compare rise time, amplitude of response, and trailing time between children and adults and found comparability in the initial and peak components of the BOLD signal. Few studies have focused on vascular contribution to the BOLD signal in children. Thomason et al. (2005) used a breath holding method with children and found greater percent change in the BOLD signal globally and in many subregions of the brain. To our knowledge, only two studies have employed ASL to study the effects of CBF development on the BOLD signal during stimulation. One activity-based study was conducted during passive visual stimulation in a sleeping infant and young children rather than school aged children (Born et al., 2002). Since school-age children and adolescents comprise the majority of subjects used in developmental neuroimaging studies (due in large part to their ability to successfully perform tasks in the MRI environment and to remain still during data collection), understanding the relationship between resting CBF levels and the BOLD signal in school-age children is particularly important. Our group has recently reported results of an ASL investigation of CBF development and BOLD signal dynamics in the auditory cortex, where we found elevated resting and activity-driven CBF in healthy children compared to adults; a concomitant age-related difference in the magnitude of the BOLD response was not detected (Moses et al., 2013). Since cortical regions reach mature CBF levels at different rates and because radionucleotide and ASL studies suggest that the trajectory of CBF development is different among various primary and secondary association cortices, it will be important to determine whether the pattern of results found in the auditory cortex are replicated in other brain regions. In the current study, we use ASL to quantify resting and activity-related CBF levels in the motor cortex during a finger-tapping task and relate these measures to BOLD data simultaneously collected in a sample of healthy, typically developing children and adults.

## MATERIALS AND METHODS

### PARTICIPANTS

A total of 28 healthy participants comprised three age groups; eight 8 year olds (*M* = 8.93 years, range = 8.12–9.97 years, one male), ten 12 year olds (*M* = 12.31 years, range = 10.73–13.51 years, four males), and ten adults (*M* = 22.42 years, range = 19.75–25.77 years, five males). These age groups were selected to target a period in childhood when CBF is elevated (8 years of age) relative to adults, a point in late childhood and early adolescence when CBF is beginning to decline toward adult levels (12 years of age), and a state of maturity as a basis of comparison. These groups are also representative of ages often studied in developmental fMRI studies. Participants were recruited from local science fairs and through advertisements in a local parent magazine. All participants were screened to exclude individuals with a history of psychological or neurological conditions, learning disabilities, premature birth, or sources of bodily metal. All participants were right-handed based on self- or parent report. Adults and parents of child participants gave informed consent and children gave informed assent in accordance with the Institutional Review Boards at San Diego State University and the University of California, San Diego.

### MOTOR TASK

The motor task consisted of tapping each of four fingers on a button box in a serial order beginning with the index and repeating the sequence throughout task periods in a block design. Participants were cued to tap at a rate of 1 Hz by a visual display projected on a screen at the foot of the scanner and viewed in a mirror placed above their eyes. All participants practiced the cued sequence on a laptop prior to the image session. A functional run began with 40 s of rest, after which 20 s of task and 40 s of rest were alternated for a total of four cycles and a total duration of 4 min 40 s per run.

### IMAGE ACQUISITION

Images were acquired on a General Electric Signa 3T whole body system, with an 8-channel receive only head coil and a body transmit coil. A 3 min resting state scan and functional runs were acquired with an ASL PICORE QUIPSS II sequence with a dual-echo spiral readout (Wong et al., 1998) using the following parameters: TR = 2 s, TI_1_ = 600 ms, TI_2_ = 1500 ms, TE_1_ = 9.5 ms, TE_2_ = 30 ms, θ = 90°, FOV = 24 cm × 24 cm, matrix size = 64 × 64, slice thickness = 6 mm. Based on localizer images acquired in three planes, five slices in the axial plane were prescribed to image the primary motor cortex. The most inferior slice was positioned to include the fundus of the central sulcus in the location of the omega-shaped “hand” region of the precentral gyrus (Yousry et al., 1997). A tagging band 100 mm thick was placed 10 mm below the most inferior imaging slice. The transit time and temporal width of the bolus were determined empirically in our previous tests of 8 and 12 year old children. For quantification of resting state CBF in absolute units, a cerebrospinal fluid (CSF) reference scan (TR = 4 s, TI_1_ = 700 ms, TI_2_ = 1400 ms, θ = 90°, FOV = 24 cm × 24 cm, matrix size = 64 × 64, TE_1_ = 9.5 ms, TE_2_ = 50 ms) and a minimum contrast scan (TR = 2 s, TI_1_ = 700 ms, TI_2_ = 1400 ms, θ = 90°, FOV = 24 cm × 24 cm, matrix size = 64 × 64, TE_1_ = 11 ms, TE_2_ = 50 ms) were collected. A high-resolution structural image was also acquired using a 3D fast spoiled gradient (FSPGR) pulse sequence (1.0 mm slice thickness; TR = 7.6 ms, TE = 2.9 ms, TI = 450 ms, θ = 12°; FOV = 25 cm x 25 cm; matrix = 256 x 256; NEX = 1). At the time of image acquisition, subjects were positioned so that their anterior and posterior commissures were aligned in a single axial plane in native space.

### PHYSIOLOGICAL DATA

To minimize physiological noise in the image data, cardiac pulse, and respiratory effort were recorded during image acquisition with a pulse oximeter (InVivo, Orlando, FL, USA) placed on the participant’s left index finger and a belt with a respiratory effort transducer (BIOPAC Systems, Goleta, CA, USA) positioned around the participant’s chest, respectively.

### DATA ANALYSIS

Data were processed and analyzed with a method previously described in our study of the auditory cortex (Moses et al., 2013). Analyses were conducted with the Analysis of Functional NeuroImages (AFNI) software platform (Cox, 1996). Two functional runs from each subject were analyzed. In each dual-echo ASL run, tag image acquisition was interleaved with control images throughout the time series. Thus from each functional run, a CBF time series was derived from the first echo with a running subtraction of each tag image from the mean of its neighboring control images. A BOLD time series was derived from the second echo images with a running subtraction of each tag image from the mean of its adjacent control images (Liu and Wong, 2005). Physiological noise was removed (Restom et al., 2006) and the 3D volumes were registered within and between runs. All subjects’ time series were screened for gross movement by visual inspection and assessed quantitatively by determining the maximum displacement between images in a time series based on the output parameters from the 3D volume registration. All data sets met the criterion of having fewer than 10% of the time points with a displacement greater than 0.75 mm (Shih et al., 2011).

A multiple regression model was used to identify voxels with task-related activation on an individual participant basis. For each participant, the two functional runs were concatenated. In separate CBF and BOLD analyses, the motor task function was the regressor of interest with six motion parameters, a baseline, and a linear trend as nuisance regressors. Subsequent analyses focused on voxels within the region of interest (ROI), described below, where multiple comparisons were control for with a Monte-Carlo simulation using AlphaSim to estimate a minimum cluster volume of 337.5 μl with a voxel-wise probability of 0.05, which corresponded to a cluster-wise activation probability of *p* ≤ 0.05. Suprathreshold clusters in the CBF activation maps were spatially contiguous with or overlapped clusters in the BOLD maps. The resultant clusters were analyzed further.

### REGION OF INTEREST ANALYSIS

The high-resolution anatomical images and the functional datasets were co-registered for localization of the task-related activation. The ROI consisted of the precentral and postcentral gyri in the left hemisphere, which corresponded to the dominant, tapping hand in all participants. These structures were delimited by manual tracing performed on the anatomical images in the axial plane. Beginning with a slice through the fundus of the central sulcus in the location of the omega-like form of the precentral gyrus (see Yousry et al., 1997) the gyri were traced successively on superior slices through the structure. To generate group activation maps that displayed the location of suprathreshold clusters, each subject’s anatomical images and functional maps were transformed into Talairach coordinates and mean CBF and BOLD maps were generated for each group.

The activated voxel clusters within the ROI yielded a mean time series for the CBF and for the BOLD data. The four task/rest cycles in each time series were averaged to produce a single task response cycle for each participant. Multiple measures were calculated from the CBF and the BOLD task response cycles. The absolute change in CBF between rest and motor activity was measured as the difference between the average signal during the initial rest period and the average signal value at the time points corresponding to the peak response, defined as the 7th–13th image acquisition time points (TRs) after the onset of the task to allow for the hemodynamic rise time. The percent of CBF signal change and the percent of signal BOLD change were calculated as the percent of signal increase from the initial rest period to the peak response period. Resting state CBF was quantified from the 3 min resting state time series in the voxels within the ROI.

### STATISTICAL ANALYSIS

Age groups (8 year olds, 12 year olds, and adults) were compared in a one-way analysis of variance (ANOVA) test for each dependent measure, mean resting CBF, absolute CBF change, percent change in CBF, and percent change in the BOLD signal during the task versus rest. Least significant difference (LSD) tests between age groups were performed to compare individual group means.

## RESULTS

**FIGURE 1:**
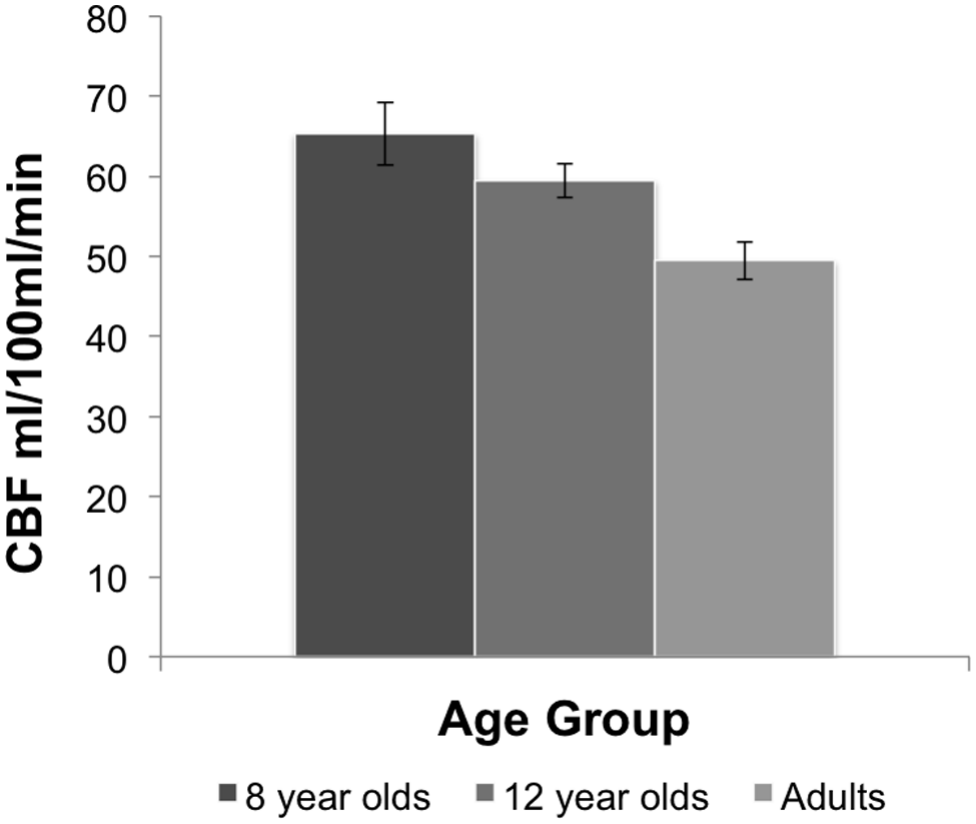
**Resting CBF in each age group.** The 8 year old and 12 year old groups display higher CBF than adults. Bars indicate the standard error.

A one-way ANOVA comparing resting state CBF between the age groups showed a main effect of age, *F*(2,25) = 8.37, *p *< 0.002. Comparisons between age groups revealed that resting state CBF was higher in the 8-year-old children (*M* = 65.30 mL/100 mL/min, *SE* = 3.90) than adults *(M* = 49.45 mL/100 mL/min, *SE* = 2.33), and in 12 year olds (*M* = 59.49 mL/100 mL/min, *SE* = 2.13) relative to adults, *p* = 0.01, based on LSD tests. No significant differences were observed between 8 and 12 year olds. **Figure 1** shows the mean resting CBF levels for each age group.

**FIGURE 2:**
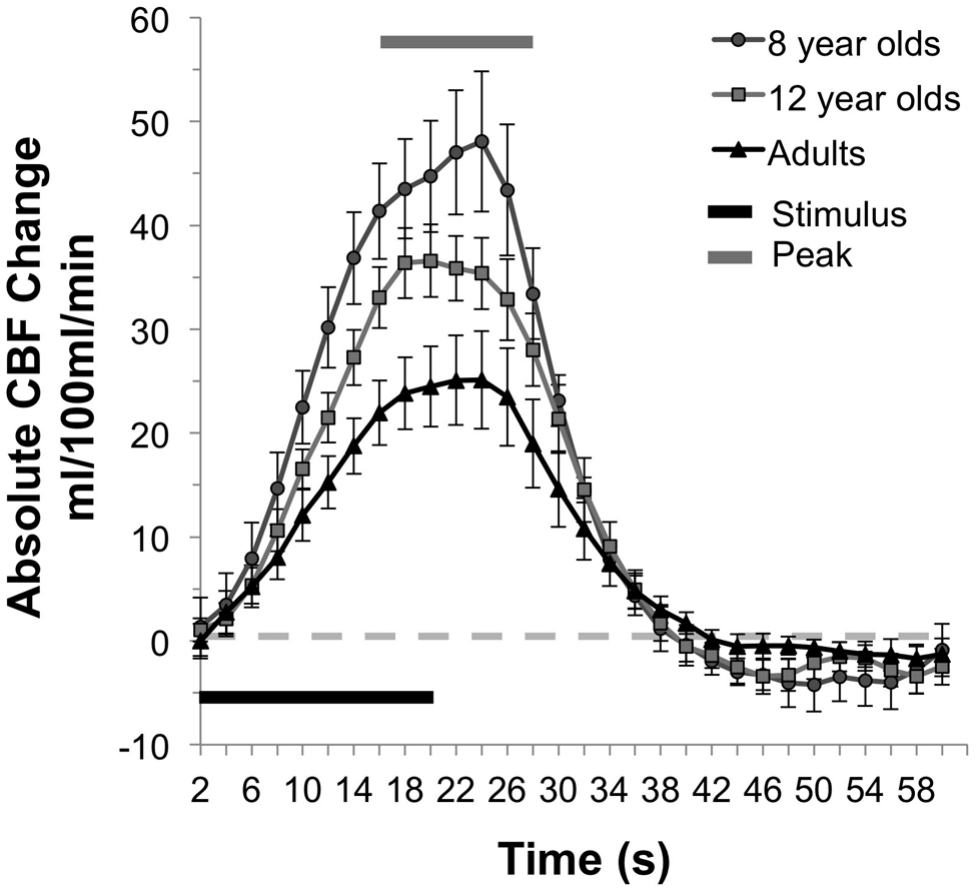
**CBF response during motor activation.** Mean time series of the absolute CBF change from baseline at each time point for each group. The single “on/off” cycle represents the mean of the four cycles within a run. In the younger children, the absolute increase in CBF response is greater than adults, and 12 year olds show a similar trend.

In addition to increased resting CBF, children had higher activity-driven CBF as well. Comparison of the absolute change in CBF between states of rest and finger-tapping in the three age groups revealed a main effect of age group, *F*(2,25) = 5.47, *p* = 0.01. Eight year olds (*M* = 45.81 mL/100 mL/min, *SE* = 5.89) had significantly greater absolute CBF change between rest and motor stimulation than adults (*M* = 24.49 mL/100 mL/min, *SE* = 4.36), *p* = 0.003. Children in the 12-year-old group showed a trend toward a greater absolute change in CBF compared to adults (*M *= 35.17 mL/100 mL/minx, *SE* = 3.30), *p* = 0.09. There were no significant differences between the 8- and 12-year-old groups. The mean times series for each of the three groups is presented in **Figure 2**, with the standard error of the mean indicated by the vertical bars and the period of stimulation and peak response (7–13 TRs after the onset of the stimulus) indicated by the horizontal bars.

**FIGURE 3:**
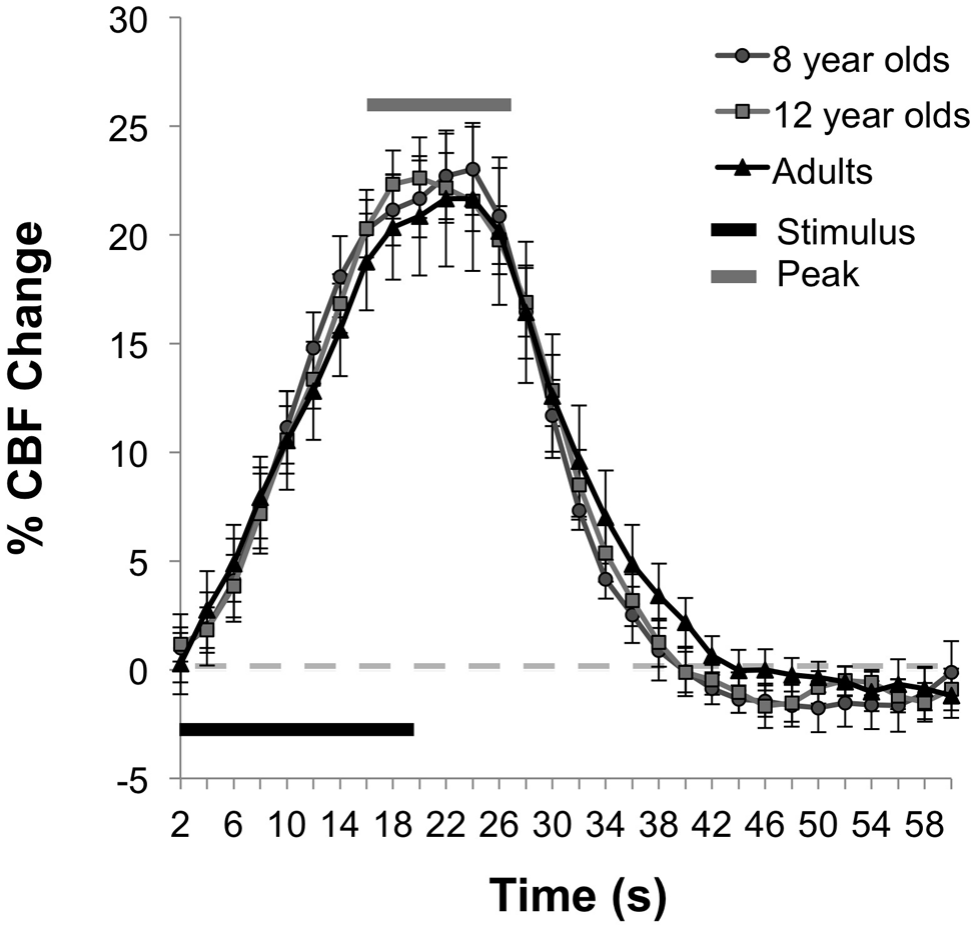
**Functional percent CBF change during motor task.** The mean time series for each age group shows the percent of CBF change during stimulation relative to baseline.

**FIGURE 4:**
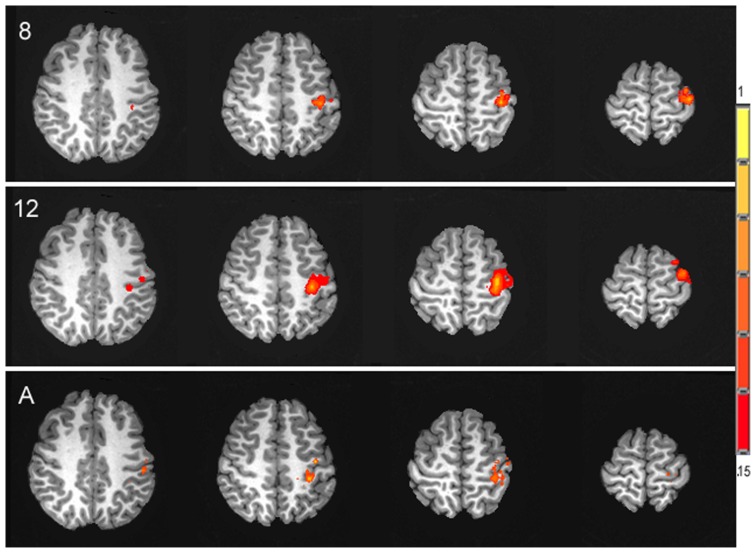
**Activation maps of the spatial distribution of the task-related CBF response in each of the three age groups, 8 year olds (8), 12 year olds (12) and adults (A), presented in the axial plane in radiological orientation.** Signal intensity is represented in the color scale.

In contrast to the age-related differences in resting and absolute CBF change, no significant differences were observed between age groups for the percent of CBF change between rest and motor stimulation, *F*(2,25) = 0.031, *p* = 0.97, (*M*s = 22.06, 21.52, 21.22) for 8 year olds, 12 year olds, and adults, respectively. The average time series of the percent of CBF change is shown for each age group in **Figure 3**. Functional activation maps presented in **Figure 4** show comparable distribution of the task-related response across the three age groups.

**FIGURE 5:**
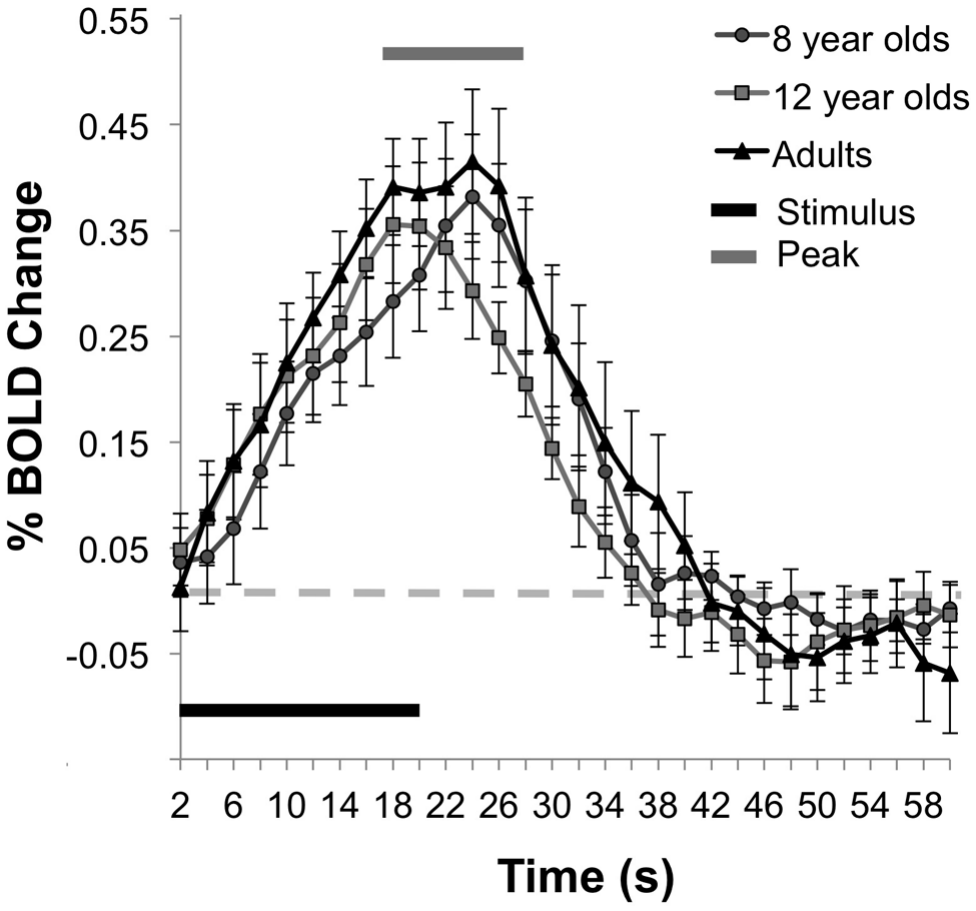
**BOLD percent signal change during activity.** The mean time series of the BOLD responses show comparability in the magnitude of the BOLD response across the age groups.

**FIGURE 6:**
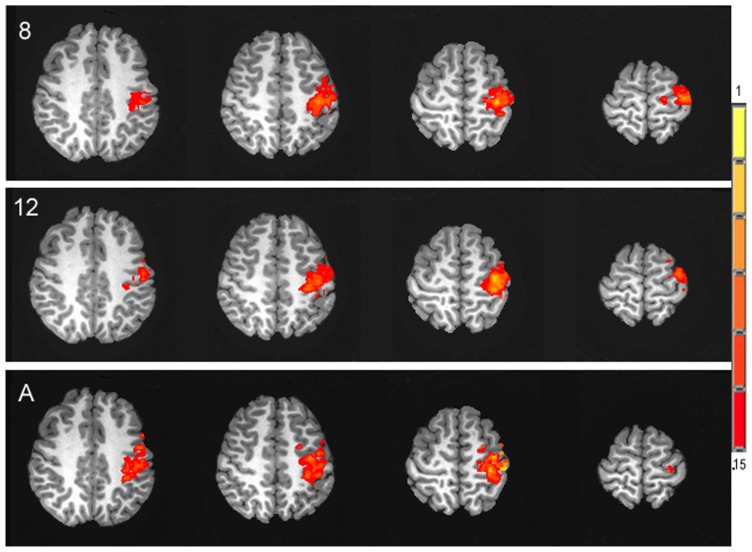
**Activation maps of the spatial distribution of the task-related BOLD response in each of the three age groups, 8 year olds (8), 12 year olds (12) and adults (A), presented in the axial plane in radiological orientation.** Signal intensity is represented in the color scale.

Similar to the percent CBF change, analysis of the percent of BOLD signal change between rest and stimulation between age groups failed to show an effect of age, *F*(2,25) = 0.44, *p* = 0.65, (*M*s for 8 year olds, 12 year olds, and adult groups are as follows, 0.33, 0.32, 0.39). **Figure 5** displays the average time series of percent BOLD change for each age group. The spatial distribution of the BOLD activation is shown for each group in **Figure 6**. *Post hoc* comparison of the descending portion of the BOLD response curve in 8 year olds, 12 year olds, and adults was performed with a repeated measures ANOVA with values of BOLD percent change from the 14th to 17th TRs after the stimulus onset. The analyses showed no significant between age group differences, *F*(2,25) = 1.01, *p* = 0.377.

## DISCUSSION

The current study was designed to investigate CBF not only during a resting state in alert children, but in response to motor stimulation and in relationship to an activity-driven BOLD response as well. As anticipated based on previous radionucleotide and ASL studies of resting state in children, this study shows age-related differences in CBF at rest. Further, these age group differences in flow are also reflected in the CBF response during performance of a motor task. The percent of increase in CBF in response to activity, however, does not differ between the age groups. Similarly the BOLD response is comparable between the three ages tested.

The changes in CBF between the two groups of children and adults reflect previous studies conducted with children in a clinical context with PET and SPECT. These studies have shown that CBF increases rapidly in the first years of life to exceed adult levels in early childhood, with gradual slowing to an adult rate during teenage years (Ogawa et al., 1989; Chiron et al., 1992; Barthel et al., 1997; Takahashi et al., 1999). The precise timing of the transition point from peak flow in the immature brain to the maturational decline is not clear from the existent literature. Studies that have examined an age span from early childhood to adulthood have often divided their broad span at the midpoint and reported differences between the younger and the older age groups. The younger groups have been capped at 5–6 years (Chiron et al., 1992), 7 years (Takahashi et al., 1999), 9 years (Ogawa et al., 1989), and 10 (Barthel et al., 1997) years of age, for example. Overall, our data concur with the patterns they observed.

Our profile of resting state CBF change from middle and late childhood to adult rates is also consistent with the small number of developmental ASL studies to date. In a large study of patients 4–78 years of age, (Biagi et al., 2007) observed greater CBF in early grade-school age children and a marked decline in CBF after 10 years of age. Wang et al. (2003b) similarly found that CBF was elevated relative to adults in a group of seven subjects 1 month to 10 years of age. Finally, a very large study of children 5–18 years of age revealed that in most brain regions, CBF decline begins between 9 and 12 years of age (Taki et al., 2011).

In contrast to the earlier neuroimaging studies of CBF in children during rest, we also investigated CBF during a condition of motor activity to see whether the flow response during an active state also differed between children and adults. Indeed, the absolute difference in CBF from rest to peak response during stimulation was higher in 8 year olds than adults. Yet, when the baseline differences in CBF were accounted for by expressing the stimulus-driven peak response as a percentage of the resting baseline, the magnitude of change was not different between any of the age groups. That is, the degree of increase in CBF response to stimulation appears to be consistent, regardless of age.

Further, acquisition of T2^*^-weighted BOLD images simultaneous with the ASL images allowed for direct comparison of CBF and BOLD responses. Interestingly, despite the elevated CBF seen at rest and during stimulation, the BOLD response did not differ between the age groups. Instead the percent of increase in the BOLD signal is fairly stable between children and adults. This finding replicates our original observation of flow and BOLD hemodynamics in the auditory cortex in response to sensory stimulation (Moses et al., 2013). Within the superior temporal auditory cortex, we also found age differences in resting CBF and in the absolute increase of CBF during stimulation, while the percent of CBF increase and the BOLD response were comparable across ages.

The absence of developmental differences in the BOLD signal, despite the high baseline, is seemingly incongruent with our understanding of the BOLD effect. The BOLD signal arises from changes in blood oxygenation that occur as a result of multiple physiological mechanisms. During rest, arterial blood delivers oxygen to the capillary beds. Oxygen is extracted and metabolized there, and the deoxygenated blood drains through the venous system. Deoxyhemoglobin is paramagnetic such that it attenuates the magnetic resonance signal. However, in association with neural activity, CBF increases, cerebral blood volume increases, and the cerebral metabolic rate of oxygen increases only modestly. As a consequence, the oxygen extraction fraction declines and a greater amount of oxygenated blood enters the veins. The net decrease of the deoxyhemoglobin lessens the local noise in the magnetic field and increases the magnetic resonance signal, which produces the BOLD effect (Ogawa et al., 1990, 1992). The mechanisms underlying the BOLD effect suggest that elevated rates of CBF in children could result in an even greater concentration of oxygenated blood in the venous system and a heightened BOLD response that reflects flow rather than cognitive strategy or neural function, as might be assumed.

Yet other aspects of the physiological state of the developing brain during childhood might explain why the relative increase in CBF in the immature brain does not seem to drive an elevated BOLD response in children as well. In children, both oxygen metabolism and glucose metabolism have been observed to be elevated. Regional cerebral metabolic rate for oxygen (rCMRO_2_) has shown a profile of an early overshoot above adult levels. rCMRO_2_ measured with PET demonstrates higher rates in early childhood (between 3 and 8 years of age) than in adulthood in many brain regions, while some sites attained adult levels between 8 and 16 years of age or later. In addition, rCMRO_2_ levels were higher in primary sensory areas than association regions (Takahashi et al., 1999). Regional cerebral metabolic rates for glucose (rCMRglu) measured with 2-deoxy-[^18^F]-fluoro-D-glucose (FDG) PET has also demonstrated an increase from the first days of life until 3–4 years of age when levels are approximately twice as high as adults. The level of rCMRglu was sustained in younger children (3–8 years of age) relative to older children (9–15 years of age) and adults (Chugani et al., 1987). Thus the relative balances of CBF with CMRO_2_and rCMRglu may be similar across age groups. A third mechanism that could play a role in offsetting high CBF is cerebral blood volume. The combination of an increase of CBV with increased CBF and a moderate increase in CMRO_2_ could lower the concentration of oxyhemoglobinin the draining veins and attenuate the BOLD signal.

In addition, the profile of an early and rapid increase in the physiological rates of CBF, rCMRO_2_ and rCMRglu, an elevated state through middle childhood and decline in adolescence is evident in structural features of the brain as well. For example synaptic density (Huttenlocher, 1990), dendritic density (Purpura, 1975), and gray matter volume (e.g., Giedd et al., 1999; Courchesne et al., 2000) all show this arching trajectory during development. At the same time, myelination is increasing steady across childhood and adolescent years. Given the overproduction and exuberance that characterizes the preadolescent brain; it is likely that the upregulation in flow and metabolic processes serves to maintain this brain state and that it declines later in concert with processes of elimination and refinement of the system that ensue. It is conceivable then that the net effect of the upregulation of multiple physiological mechanisms results in BOLD response that resembles an adult’s. Thus it appears that there may be conservation of the magnitude of the BOLD signal.

Examination of the interplay between cerebrovascular and cerebrometabolic processes and their role in the BOLD effect has been an active area of research in the mature brain. In contrast, the question of whether or not there are developmental differences in these processes or in the way these processes interact has been left largely unexamined in the immature brain (see Harris et al., 2011 for a review of potential physiological confounds to the BOLD response in the developing brain). The few studies that have examined properties of the BOLD signal alone in the developing brain of alert children present findings that are complementary to the current study. Richter and Richter (2003) and Wang et al. (2003b) found that the timing of the rise the BOLD signal and the peak or magnitude of the BOLD response did not change with age. Kang et al. (2003) compared the time course of the BOLD response in children 7–8 years of age with adults in a sensorimotor task and found comparability between-groups. Further in conditions of a brief or sustained stimulus, the BOLD response remains comparable between children and adults (Wenger et al., 2004).

Thomason et al. (2005) specifically examined vascular dynamics in relationship to the BOLD signal in children 7–12 years of age with a breath holding paradigm. Resting state breath holding increases CO_2_ levels in blood (hypercapnia) and results in an increase of CBF, without a corresponding gain in task-induced CMRO_2_. With this approach, they found the percent of BOLD signal change between rest and breath holding was greater in children than adults in multiple brain regions including the motor cortex. That is, when CBF was driven up further in children by breath holding in the absence of increases in CMRO_2_, the BOLD response increased. This is consistent with our findings and the possibility that elevations in both CBF with CMRO_2_ yield a BOLD response similar to adults’. Further, studies of age-dependent changes in cerebrovascular coupling later in life, between young and older adulthood, provide complementary findings. In the aging brain, Kannurpatti et al. (2011) compared parameters of the BOLD response, including amplitude change in a finger-tapping task, in younger and older adults. Comparison of the amplitude of the BOLD response alone yielded no group differences. However, they also measured physiological indices reflecting vascular components, the hemodynamic response during breath holding and the amplitude of low frequency variations in the resting state BOLD signal [resting state fluctuation of amplitude (RSFA)] and scaled the original BOLD signal by each of these measures. When vascular contributions were accounted for in this manner, both forms of scaling revealed group differences in the remaining, presumably neural component of the signal. Recent analyses of RSFA in a large-scale study of 335 adults 20–85 years of age have shown region-specific, age-related changes in RSFA. In addition, when the BOLD signal is scaled by RSFA, some age-related differences are no longer supported (Tsvetanov et al., 2013). Thus studies of development and aging both underscore the importance of understanding and accounting for vascular dynamics in measuring and interpreting the BOLD signal as an indirect measure of neural activity.

This study examined CBF during both states of rest and activation, as well as its relationship to the BOLD signal in the developing brain. This investigation reveals greater CBF in the motor cortex in response to finger-tapping and at rest in children compared to adults. Although flow is greater in the immature brain in both states, the percent of CBF increase during activity and the percent signal increase in the BOLD signal remain stable between 8 year olds, 12 year olds and adults. These finding mirror our results in a previous study of CBF and BOLD hemodynamics in the auditory cortex of children and adults. Thus, this set of findings generalizes between these different brain regions and these sensory and motor systems. Importantly this study demonstrates that while the BOLD response of children resembles the response of adults in terms of the magnitude of the signal, the underlying CBF, and likely other physiological mechanisms that contribute to the signal, are different in developing and mature brains.

## Conflict of Interest Statement

The authors declare that the research was conducted in the absence of any commercial or financial relationships that could be construed as a potential conflict of interest.
